# Synthesis of Zn-based 1D and 2D coordination polymer nanoparticles in block copolymer micelles†

**DOI:** 10.1039/d0na00334d

**Published:** 2020-09-08

**Authors:** Christoph Göbel, Gerald Hörner, Andreas Greiner, Holger Schmalz, Birgit Weber

**Affiliations:** Department of Chemistry, Inorganic Chemistry IV, Universität Bayreuth Universitätsstraße 30 95447 Bayreuth Germany weber@uni-bayreuth.de; Department of Chemistry, Macromolecular Chemistry II, Keylab Synthesis and Molecular Characterization, Bavarian Polymer Institute, Universität Bayreuth Universitätsstraße 30 95447 Bayreuth Germany holger.schmalz@uni-bayreuth.de

## Abstract

Nanoparticles of the 1D and 2D coordination polymers [Zn(OAc)_2_(bipy)]_*n*_ and [Zn(TFA)_2_(bppa)_2_]_*n*_ were prepared, employing polystyrene-*block*-poly(4-vinylpyridine) diblock copolymers with different weight fractions of the 4-vinylpyridine (4VP) block and comparable overall molecular weights of *M*_n_ ≈ 155 kg mol^−1^ as template (SV-15 and SV-42 with 15 and 42 wt% 4VP, respectively). [Zn(OAc)_2_(bipy)]_*n*_ nanoparticles were successfully synthesised within the 4VP core of SV-42 micelles, showing a core size of *D*_core_ = 47 ± 5 nm and a hydrodynamic diameter of *D*_h_ = 157 ± 46 nm, determined by transmission electron microscopy (TEM) and dynamic light scattering (DLS). The crystallinity of the composite is quite low, showing only low intensity reflexes in the powder X-ray diffraction (PXRD) pattern with the highest particle load. No indications for larger microcrystals were detected by scanning electron microscopy (SEM), proving the successful integration of the coordination polymer nanoparticles within the micellar cores. Nanocomposites of the 2D coordination network [Zn(TFA)_2_(bppa)_2_]_*n*_ were synthesised using both diblock copolymers. The particle core sizes (from TEM) and hydrodynamic diameters (from DLS) correlate with the 4VP fraction of the micelles, resulting in *D*_core_ = 46 ± 6 nm for SV-42 and 15 ± 2 nm for SV-15 and *D*_h_ = 340 ± 153 nm and 177 ± 57 nm, respectively. The successful synthesis was proven by PXRD and SEM images, confirming the absence of larger crystallites. Hence, it is possible to synthesise nanocomposites of Zn-based 1D and 2D coordination polymers by a direct approach utilizing diblock copolymer micelles as template.

## Abstract 2

Synthesis of 2D coordination network nanoparticles using block copolymer micelles as template.

## Graphical abstract

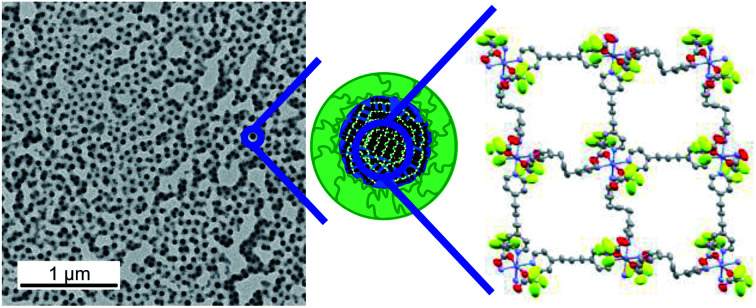

## Introduction

Devices built up from functional molecular materials are an interesting approach to realize new functionalities for new fields of applications. Examples for promising molecule-based systems are porous coordination networks (MOFs, metal organic frameworks), Prussian blue based materials or molecular magnetic materials including spin crossover coordination polymers.^1–9^ Nanoparticles and nanocomposites of such materials are often considered to play a key role in future device engineering.^10–15^ However, the synthesis of well-defined, stable nanoparticles or nanocomposites of molecule-based materials is a highly demanding task, as a wide range of techniques successfully used for solid state materials (*e.g.* the reduction of metal salts^16–20^ or the hydrothermal synthesis^21–23^) are inapplicable. For molecular materials, some synthetic procedures like the inverse micelle technique^24–28^ or micro-fluidic approaches using fast precipitation^29–31^ have already been established to achieve that task. However, each new material has its needs regarding the reaction conditions (*e.g.* reaction temperature, solvent, reactant solubility, air or moisture sensitivity). Furthermore, some of the approaches have limitations regarding the size limits that can be reached. This makes a fine-tuning of the reaction conditions indispensable to not only achieve a successful synthesis of the nanomaterial of the desired size, but also to preserve the desired properties. Furthermore, some synthesis procedures have been proven more suitable for the formation of functional materials than others, because they allow for example the even distribution of the nanomaterial or nanocomposite on surfaces or prevent the aggregation of the formed nanoparticles.^32,33^

Nanoparticles of 2D^34–36^ or 3D^24,37,38^ coordination networks (CNs) have been prepared with a wide range of bridging ligands and metal ions. However, the formation of 2D and 3D CN nanoparticles directly in the core of block copolymer micelles is quite rare. To the best of our knowledge, only 6 examples of 2D or 3D CN nanoparticles formed in a polymer matrix can be found in the literature.^39–44^ A more commonly used technique is the immobilization of pre-formed nanoparticles in block copolymer micelles or polymer matrices (bulk polymers, gels, *etc.*),^45–55^ in some cases even size-selective employing polymer cages.^56^

We have previously shown that the use of polystyrene-*block*-poly(4-vinylpyridine) (PS-*b*-P4VP) diblock copolymers (BCPs) is ideal for the size-controlled synthesis of 1D Fe(ii) spin crossover (SCO) coordination polymer (CP) nanoparticles with core sizes of 16 ± 2 nm and 48 ± 4 nm. It was possible to retain the SCO properties with hysteresis at both particle sizes. Thermal treatment of the 16 nm particles triggers a confined crystallization of the NPs leading to SCO properties comparable to those of the bulk material.^57,58^ In other cases, the synthesis in confinement results in different morphologies for NPs and bulk material and therefore different SCO properties.^59^

Herein, we report the successful adaptation of our general synthetic concept to a completely new type of CPs and for the first time to a 2D CN to illustrate its general applicability. The double-stranded 1D CP [Zn(OAc)_2_(bipy)]_*n*_ ^60^ (bipy = 4,4′-bipyridine) and the layer-like 2D CN [Zn(TFA)_2_(bppa)_2_]_*n*_ ^61^ (TFA = trifluoroacetic acid, bppa = 1,3-di(4-pyridyl)propane) were used for the formation of Zn-CP/CN-BCP nanocomposites. The nanocomposites were synthesised using two PS-*b*-P4VP diblock copolymers (SV-15 and SV-42) as templates, which have an almost identical molecular weight but differ in the weight fraction of the 4VP blocks (see Table 1).

**Table 1:** Overview of the used BCPs in this work

BCP	*M*_n_ a [g mol^−1^]	*Đ* b	PS : P4VP^c^ [w/w]	*D*_core_ d [nm]	*D*_h_ e [nm]
SV-15	154 000	1.02	85 : 15	15 ± 2	75 ± 28
SV-42	157 000	1.09	58 : 42	45 ± 5	125 ± 34

a Calculated from proton nuclear magnetic resonance (^1^H NMR) measurements, using the molecular weight of the PS precursor measured by matrix-assisted laser desorption-time of flight mass spectrometry (MALDI-ToF MS).

b From gel permeation chromatography (GPC) in *N*,*N*-dimethylformamide using narrowly distributed PS standards for calibration.

c Calculated from ^1^H NMR measurements.

d Core diameters of empty BCP micelles, see Fig. S1 for TEM images and core size distributions.

e Hydrodynamic diameters of the empty BCP micelles, see Fig. S2 for DLS measurements.

## Results and discussion

### Synthetic procedures

**Scheme 1:**
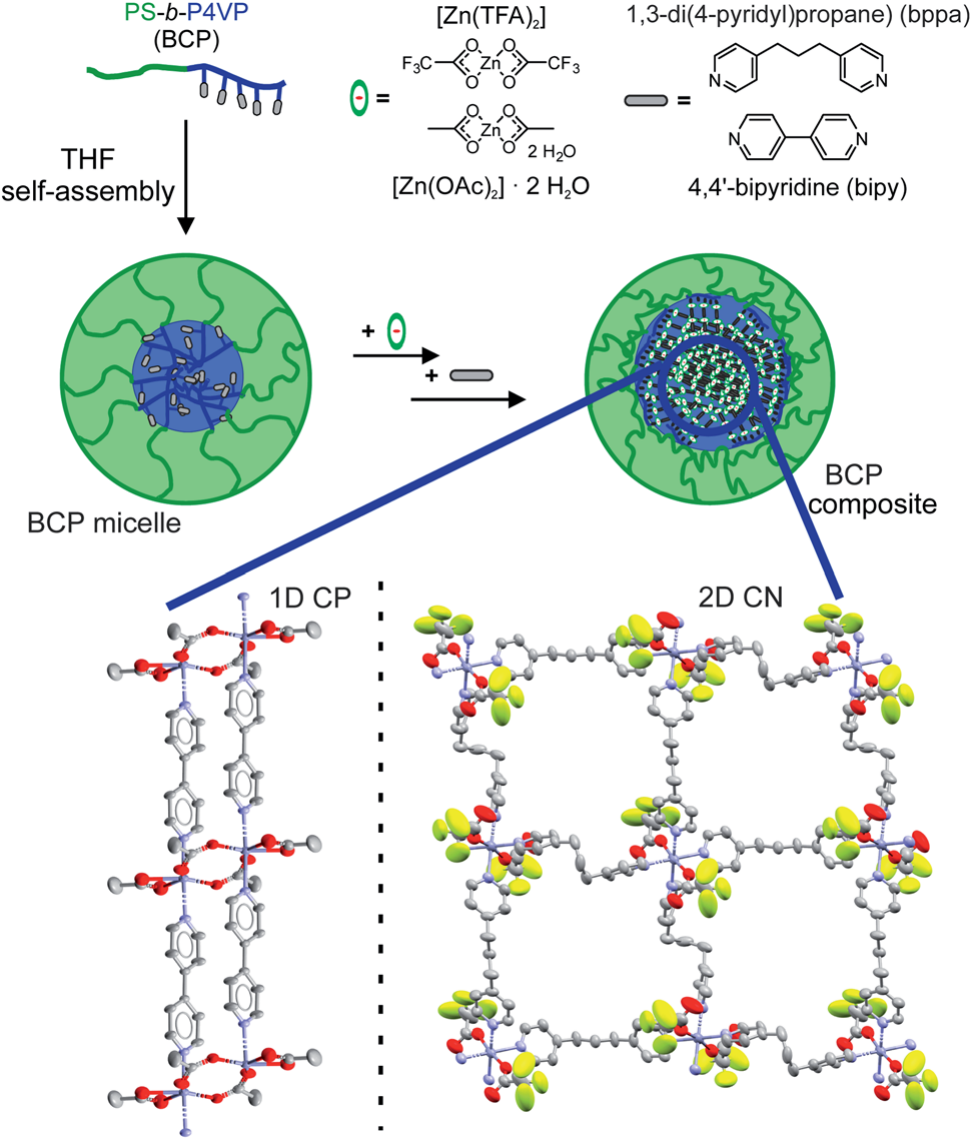
Synthetic approach for the preparation of Zn-based CP-BCP nanocomposites. An excerpt of the crystal structures of both CP bulk materials is given below (left: [Zn(OAc)_2_(bipy)]_*n*_, right: [Zn(TFA)_2_(bppa)_2_]_*n*_).

The synthesis procedure was adapted from the literature and adjusted to the requirements of the Zn-based CPs (Scheme 1).^58^ Dissolving the diblock copolymer in THF leads to the formation of BCP micelles due to the significantly lower solubility of the P4VP block compared to the PS block. Thus the less-soluble P4VP core, where the synthesis of the NPs takes place, is surrounded by soluble PS corona chains. The nanocomposite samples containing the 1D CP [Zn(OAc)_2_(bipy)]_*n*_ were synthesised employing SV-42 diblock copolymer micelles in THF (Table 2). The Zn(ii) precursor [Zn(OAc)_2_]·2H_2_O was added and the solution was refluxed for 1 h. Subsequently, the solution was cooled down, the bridging ligand bipy was added and the solution was refluxed again for 1 h. At this point, the synthesis can be stopped by removal of the solvent *via* rotary evaporation (sample 1; 1 cycle) or [Zn(OAc)_2_]·2H_2_O and bipy can be added simultaneously up to 4 more times (samples 2–4; 3–5 cycles). The resulting light-yellow solids were dried *in vacuo*.

**Table 2:** Overview of the synthesised nanocomposites

Sample	CP/CN	BCP	Cycles	*D*_core_ a [nm]	*D*_h_ b [nm]
1	[Zn(OAc)_2_(bipy)]_*n*_	SV-42	1	50 ± 4	141 ± 48
2			3	47 ± 4	155 ± 42
3			4	47 ± 4	152 ± 41
4			5	47 ± 5	157 ± 46
5	[Zn(TFA)_2_(bppa)_2_]_*n*_	SV-15	1	13 ± 1	139 ± 39
6		SV-42	1	49 ± 4	160 ± 46
7		SV-15	2	15 ± 2	177 ± 57
8		SV-42	2	46 ± 6	340 ± 153

a Core diameters of the nanocomposite particles (determined by TEM).

b Hydrodynamic diameters of the nanocomposite particles (determined by DLS).

The BCPs SV-15 and SV-42 were used for the synthesis of nanocomposites containing the 2D CN [Zn(TFA)_2_(bppa)_2_]_*n*_ (Table 2). Here, the synthesis protocol had to be adapted due to the very low solubility of the desired 2D CN. The respective BCPs were dissolved under reflux in THF to trigger the self-assembly to micelles, [Zn(TFA)_2_]·H_2_O was added and the mixture was heated to reflux for 1 h to initiate the coordination of the zinc(ii) precursor at the pyridine units in the P4VP core of the micelle.

To avoid a precipitation of the CN and to decelerate its formation, the bridging ligand bppa was dissolved in THF and added dropwise to the reaction solution over 15 min, followed by a 1 h reflux. The solvent was removed by rotary evaporation and subsequent drying *in vacuo* to yield light-yellow samples 5 and 6 (1 cycle each). The reaction procedure can be repeated to yield samples 7 and 8 (2 cycles each) with a higher complex loading. The formation of nanocomposites with higher cycle counts (>2) was tested, but the formation of microcrystals was observed by SEM (see Fig. S3†). Therefore, no further addition of reactants was conducted after the second addition of bppa (for experimental details see Experimental section).

### Characterisation of nanocomposites

In total, eight different nanocomposites have been synthesised (Table 2), of which four contain the 1D CP [Zn(OAc)_2_(bipy)]_*n*_ (samples 1–4) and another four the 2D CN [Zn(TFA)_2_(bppa)_2_]_*n*_ (samples 5–8). All nanocomposite materials were characterised by transmission electron microscopy (TEM) and dynamic light scattering (DLS) to evaluate the particle sizes in the dry state and in dispersion. Furthermore, the nanocomposites were analysed by elemental analysis (C, H, N), infrared spectroscopy (IR), powder X-ray diffraction (PXRD), and scanning electron microscopy (SEM). IR measurements were supported by computational calculations.

### [Zn(OAc)_2_(bipy)]_*n*_ nanocomposites

**Fig. 1:**
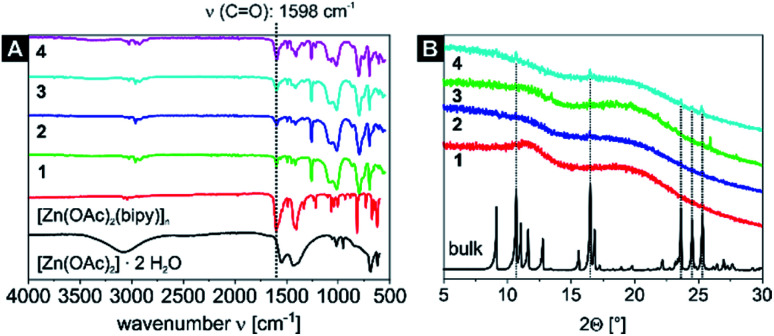
IR spectra of the starting material [Zn(OAc)_2_]·2H_2_O, the CP [Zn(OAc)_2_(bipy)]_*n*_ and the four [Zn(OAc)_2_(bipy)]_*n*_ nanocomposite samples 1–4 (A) and a comparison of the PXRD patterns of the bulk material [Zn(OAc)_2_(bipy)]_*n*_ and the [Zn(OAc)_2_(bipy)]_*n*_ nanocomposite samples 1–4 (B). PXRD reflexes that correlate with the bulk material are marked with a dashed line.

IR measurements of the starting material [Zn(OAc)_2_]·2H_2_O, the bulk material [Zn(OAc)_2_(bipy)]_*n*_ and the samples 1–4 are displayed in Fig. 1A. The nanocomposites show a characteristic band at 1598 cm^−1^, which increases in intensity relative to other bands when higher cycle counts are reached. This is in excellent agreement with the spectrum of independently synthesised bulk [Zn(OAc)_2_(bipy)]_*n*_, which features a band at 1600 cm^−1^. Thus, this band can be safely assigned to the C=O stretching mode of the neat CP. Peak assignment in the fingerprint area between 1400 cm^−1^ and 1800 cm^−1^ proved valuable to identify the nature and purity of the nanocomposites, which was further supported by numerical frequency calculations of optimized model structures. The CP was approximated as binuclear [Zn_2_(OAc)_4_(py)_4_], whereas the H-bond network of the precursor was taken into account in pentanuclear [Zn(OAc)_2_(OH_2_)] × 4 [Zn(OAc)_2_(OH_2_)] (see Experimental section for computational details, animations of diagnostic modes are given in the ESI,† anim_1–6). In fact, the calculated C=O stretching mode in the CP model [Zn_2_(OAc)_2_(py)_2_]_*n*_ is located at 1601 cm^−1^, almost identical to samples 1–4 and the bulk material. This is a distinct difference to the C=O band of the precursor [Zn(OAc)_2_]·2H_2_O, which is experimentally found at 1549 cm^−1^ (computed value: 1534 cm^−1^). The formation of single-stranded [Zn(OAc)_2_(bipy)]_*n*_ can be similarly ruled out, as C=O based stretching modes computed for the model [Zn(OAc)_2_(py)_2_] are predicted at 1500 cm^−1^, proving the successful synthesis of the 1D CP in the P4VP core of the SV-42 micelles.

Further proof is given by the PXRD patterns of the samples 1–4. Samples 1–3 are highly amorphous as indicated by the powder diffraction patterns. Only sample 4 with five reaction cycles shows five reflexes that also correspond to the dominant reflexes of the bulk material (Fig. 1B) indicating a successful formation of the CP inside the micellar core.

**Fig. 2:**
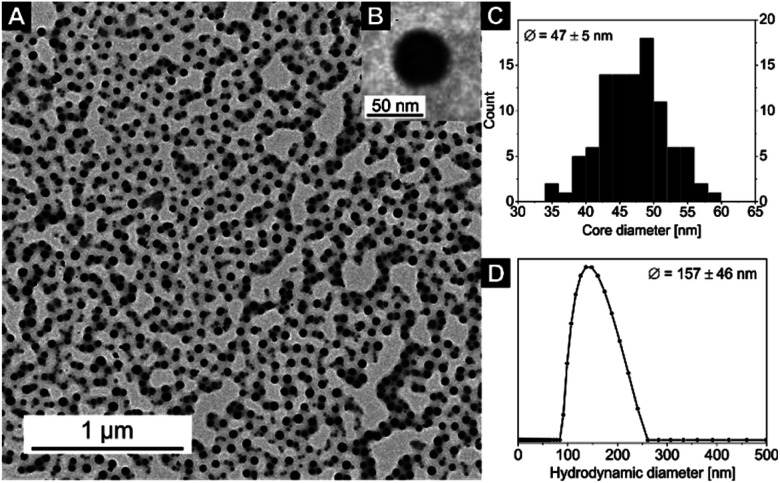
(A) TEM image of sample 4, the cores of the micelles with the embedded [Zn(OAc)_2_(bipy)]_*n*_ CP nanoparticles appear black, (B) an inset with a zoom on a single nanoparticle, (C) core size distribution *D*_core_ (from TEM) and (D) hydrodynamic diameter distribution *D*_h_ (from DLS) of the nanocomposite sample 4 (for DLS autocorrelation function see Fig. S6†).

Exemplary for all nanocomposites with the [Zn(OAc)_2_(bipy)]_*n*_ CP, the TEM and DLS measurements of sample 4 are displayed in Fig. 2. The corresponding core diameter and hydrodynamic diameter of all samples are summarized in Table 2. The DLS measurement shows narrowly distributed nanocomposite particles with a hydrodynamic diameter of *D*_h_ = 157 ± 46 nm. As the electron-rich [Zn(OAc)_2_(bipy)]_*n*_ CP is incorporated inside the micelle core of the BCP, only the core of nanocomposite particles is clearly visible in TEM, resulting in notably smaller diameters compared to DLS. The TEM image of sample 4 shows spherical particle cores with a core size of *D*_core_ = 47 ± 5 nm (Fig. 2). In line with the results for other coordination polymers reported so far,^57–59^ particles core sizes and hydrodynamic diameters of samples 1–3 are nearly identical and slightly increased compared to the empty template. Respective data of all samples confirming these results can be found in Fig. S4 and S5† together with the autocorrelation function of sample 4 (Fig. S6†).

The samples 1–4 were also characterised by SEM, revealing the absence of microcrystals on the sample surface (Fig. S7†). Thus, the CP is regioselectively formed inside the cores of the BCP micelles.

### [Zn(TFA)_2_(bppa)_2_]_*n*_ nanocomposites

**Fig. 3:**
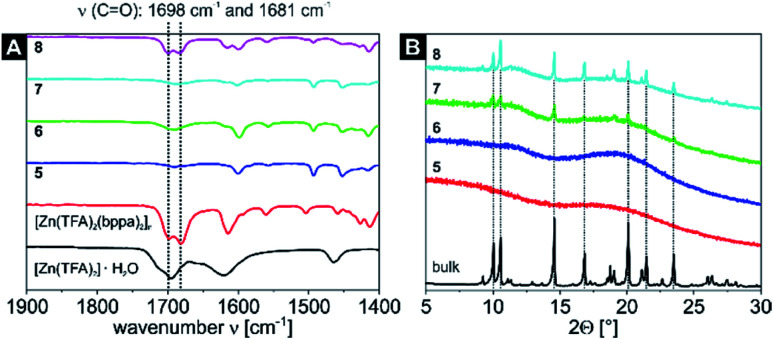
Detailed view on the spectral range of 1900 cm^−1^ to 1400 cm^−1^ of the IR spectra of the starting material [Zn(TFA)_2_]·H_2_O, the CN [Zn(TFA)_2_(bppa)_2_]_*n*_ and the four [Zn(TFA)_2_(bppa)_2_]_*n*_ nanocomposite samples 5–8 (A). The complete spectra can be found in Fig. S8.† Comparison of the PXRD patterns of the bulk material [Zn(TFA)_2_(bppa)_2_]_*n*_ and the [Zn(TFA)_2_(bppa)_2_]_n_ nanocomposite samples 5–8 (B). Most intense PXRD reflexes that correlate with the bulk material are marked with a dashed line.

IR measurements were also performed for the four nanocomposites containing the [Zn(TFA)_2_(bppa)_2_]_*n*_ CN (samples 5–8, Fig. 3A and S8†) and were compared to the starting material [Zn(TFA)_2_]·H_2_O and the bulk material [Zn(TFA)_2_(bppa)_2_]_*n*_. The starting material shows a C=O band at 1695 cm^−1^ with a shoulder at 1715 cm^−1^, whereas the bulk CN shows two characteristic bands in the range of C=O vibrations at 1698 cm^−1^ and 1681 cm^−1^. Computation of a truncated mononuclear model of the CN, [Zn(TFA)_2_(py)_4_], similarly gives two bands at 1668 cm^−1^ and 1662 cm^−1^. For samples 5–7 only one band was detected at 1690 cm^−1^, which is exactly between the two bands of the bulk CN. For sample 8 two bands for the CN were determined at 1699 cm^−1^ and 1684 cm^−1^, being in good agreement with the bulk material. Again, a relative increase in intensity of the carbonyl band is detectable with higher cycles. Thus, it was possible to incorporate the 2D CN into the 4VP cores of both micellar templates (SV-15 and SV-42). In line with the PXRD results of the [Zn(OAc)_2_(bipy)]_*n*_ CP nanocomposites, the samples 5 and 6 (one loading cycle) are completely amorphous as represented by the diffraction patterns. Nevertheless, samples 7 and 8 (two loading cycles) already show some reflexes at positions that match with the bulk material, indicating the successful formation of the desired CN inside the BCP micelles (Fig. 3B).

**Fig. 4:**
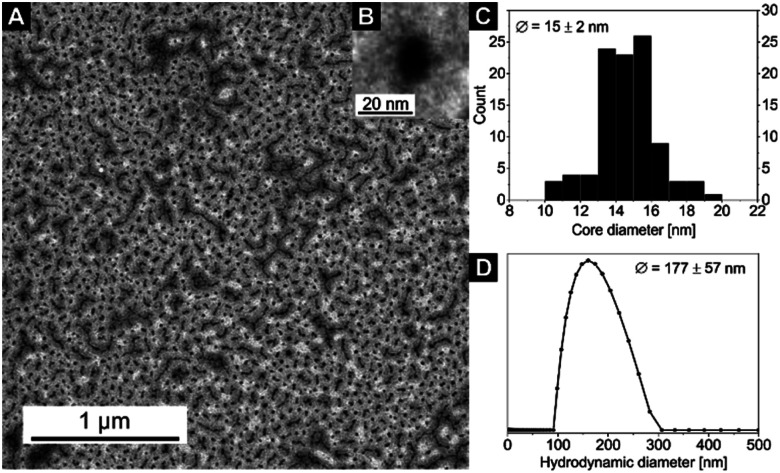
(A) TEM image of sample 7. (B) An inset with a zoom on a single nanoparticle. (C) Core size distribution *D*_core_ (from TEM) and (D) hydrodynamic diameter distribution *D*_h_ (from DLS) of nanocomposite sample 7 (for DLS autocorrelation function see Fig. S11†).

**Fig. 5:**
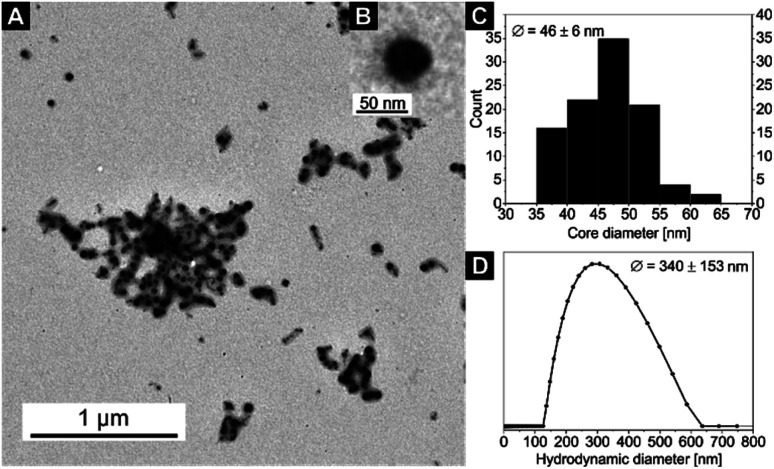
(A) TEM image of sample 8. (B) An inset with a zoom on a single nanoparticle. (C) Core size distribution *D*_core_ (from TEM) and (D) hydrodynamic diameter distribution *D*_h_ (from DLS) of sample 8 (for DLS autocorrelation function see Fig. S11†).

Particle sizes of the nanocomposites were also analysed by TEM and DLS (Fig. 4, 5; S9–S11†). While sample 5 only shows spherical particles with core sizes of *D*_core_ = 13 ± 1 nm (Fig. S9†), sample 7 shows spherical particles which, however, tend to form chain-like aggregates (Fig. 4). This behaviour was also observed in other samples of the same nanocomposite (Fig. S12†). In fact, the formation of spherical particles rather than worm-like micelles in THF would be expected in THF due to the low 4VP fraction of the utilised SV-15 diblock copolymer.^62,63^ The presence of the anisotropic 2D CN together with the limited space available in the P4VP core of the highly asymmetric SV-15 BCP micelles (*D*_core_ = 15 ± 2 nm, *D*_h_ = 177 ± 57 nm for sample 7) could trigger the formation of chain-like structures, even at comparably low 4VP fractions. This may be an effect that occurs during drying of the sample on the TEM grid, since the hydrodynamic diameter distribution of sample 7 is rather narrow (Fig. 4C) and *D*_h_ is only slightly increased compared to that of sample 5 (Table 2).

The particle core sizes of samples 6 (*D*_core_ = 49 ± 4 nm, Fig. S9†) and 8 (*D*_core_ = 46 ± 6 nm, Fig. 5) are in good agreement with the core sizes of samples 1–4 (*D*_core_≈ 47–50 nm, Fig. 2 and S4†), which were synthesised using the same BCP (SV-42, Table 2). This underlines the fact that the BCP determines the size of the nanocomposites. Again, the formed nanocomposite particles tend to form chain-like structures for sample 8 (2 loading cycles), as observed by TEM. Another interesting phenomenon arises upon comparing the DLS measurements of samples 6 and 8. While the average hydrodynamic diameter of sample 6 was determined to *D*_h_ = 160 ± 46 nm (Fig. S10†), which correlates well with the hydrodynamic diameters of the samples 1–4, the average hydrodynamic diameter of sample 8 is more than twice as large (*D*_h_ = 340 ± 153 nm, Fig. 5) and the hydrodynamic diameter distribution is significantly broadened. This might point to the presence of chain-like (worm-like) structures already in solution. The shorter soluble PS blocks in the corona of SV-42 micelles might be less efficient in shielding the highly anisotropic CN in the micellar core and, thus, favouring the formation of chain-like structures. This assumption is supported by the fact that in Fig. 5A individual spherical CN nanoparticles can be recognized in the chain-like micellar structures.

**Fig. 6:**
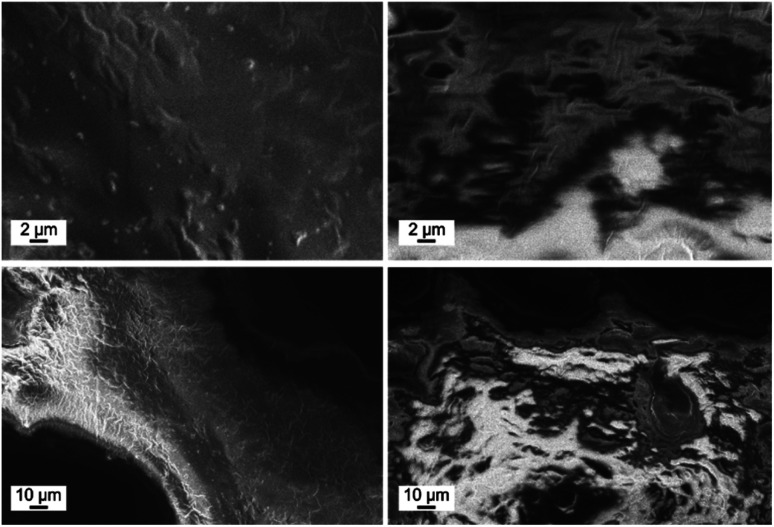
SEM images of sample 7 (left) and sample 8 (right) at two different magnifications. No microcrystals can be observed on the polymer surface for both samples.

The SEM images for the samples 5–8 (Fig. 6, and S13†) show the absence of microcrystals at the surface of the nanocomposites, proving that the 2D CN is incorporated inside the P4VP cores of the BCP micelles. However, if samples with more than 2 reaction cycles were synthesised, the very low solubility of the 2D CN [Zn(TFA)_2_(bppa)_2_]_*n*_ led to a fast precipitation of the CN, thus, resulting in the formation of microcrystals on the polymer surface and in the reaction solution. Consequently, the formation of truncated cuboctahedron crystals on the nanocomposite surface was observed by SEM (Fig. S3†).

## Conclusions

The synthesis of well-defined 1D, 2D and 3D coordination polymer (CP) and network (CN) nanoparticles is highly challenging. Self-assembled polymeric micelles derived from block copolymers (BCPs) that offer coordination sites inside the micellar core may be an elegant and generally applicable concept for the direct synthesis of these CP and CN nanoparticles (NPs). We were able to show that our established synthetic approach can be adapted to other 1D CP like the double-stranded [Zn(OAc)_2_(bipy)]_*n*_ and more importantly to the 2D CN [Zn(TFA)_2_(bppa)_2_]_*n*_. Employing micelles of the BCP SV-42 as template it was possible to achieve spherical NPs of the 1D CP [Zn(OAc)_2_(bipy)]_*n*_ and the 2D CN [Zn(TFA)_2_(bppa)_2_]_*n*_ with nanocomposite core sizes of *D*_core_ = 47 ± 5 nm and *D*_core_ = 46 ± 6 nm, respectively. The average hydrodynamic diameter was determined to *D*_h_ = 157 ± 46 nm for the [Zn(OAc)_2_(bipy)]_*n*_ and to *D*_h_ = 340 ± 153 nm for the [Zn(TFA)_2_(bppa)_2_]_*n*_ nanocomposites. Moreover, even smaller composite NPs of the 2D CN [Zn(TFA)_2_(bppa)_2_]_*n*_ were successfully prepared in SV-15 micelles, having a core size *D*_core_ as small as 15 ± 2 nm and a hydrodynamic diameter of *D*_h_ = 139 ± 39 nm. No microcrystals were found on the nanocomposite surface as proven by SEM measurements. The crystallinity of the nanocomposite samples increases with the loading cycles, showing characteristic reflexes in the PXRD at positions identical to the bulk materials. Since it was possible to synthesise NPs of the double-stranded 1D CP [Zn(OAc)_2_(bipy)]_*n*_ and particularly the 2D CN [Zn(TFA)_2_(bppa)_2_]_*n*_, we are convinced that our synthetic approach can be adapted to a wide range of other 1D, 2D, and even 3D CP and CN nanoparticles, which will be investigated in future work.

## Experimental section

### Materials

4,4′-Bipyridine (bipy, 98%), 1,3-di(4-pyridyl)propane (bppa, 98%) and [Zn(OAc)_2_]·2H_2_O (97+%) were obtained from Alfa Aesar and used as received. For the synthesis of [Zn(TFA)_2_]·H_2_O, zinc oxide (ZnO, 99.9%) from Sigma Aldrich, trifluoroacetic acid (TFA, 99%) from Alfa Aesar and ethanol (EtOH, p.a., Fisher Chemical) were used as received. Tetrahydrofuran (THF, p.a.) was obtained from Fisher Chemical and used as received in the synthesis of the coordination polymers and the nanocomposites.

The two polystyrene-*block*-poly(4-vinylpyridine) diblock copolymers (SV-15 and SV-42) were synthesised by sequential anionic polymerization of styrene and 4-vinylpyridine according to our previously published method.^57^

For gel permeation chromatography (GPC) in *N*,*N*-dimethylformamide (DMF) with lithium bromide (5 g L^−1^), GRAM columns (300 × 8 mm, 10 μm particle size, PSS Mainz) with 100 and 3000 Å pore sizes were used. The samples were measured on a SEC 1260 Infinity system (Agilent Technologies) at a flow rate of 0.5 mL min^−1^ at 23 °C, using a refractive index detector (Agilent Technologies). The calibration was done with narrowly distributed polystyrene standards (PSS calibration kit) and toluene (HPLC grade) was used as internal standard.

MALDI-ToF MS (matrix-assisted laser desorption/ionization time-of-flight mass spectrometry) measurements were performed on a Reflex III (Bruker) equipped with a N_2_ Laser (*λ* = 337 nm). An acceleration voltage of 20 kV was used in linear mode and the samples were prepared according to the dried droplet method. Matrix (*trans*-2-[3-(4-*tert*-butylphenyl)-2-methyl-2-propenylidene]malononitrile, DCTB, 10 g L^−1^ in THF), analyte (10 g L^−1^ in THF) and salt (silver trifluoroacetate, 10 g L^−1^ in THF) were dissolved and mixed in the ratio of 20 : 5 : 1 and 0.5 μL of the mixture was placed and dried on the target plate.

^1^H-NMR spectra were acquired with a Bruker Ultrashield 300 spectrometer using CDCl_3_ as solvent.

Transmission electron microscopy (TEM) was conducted on a Zeiss CEM902 electron microscope (Zeiss, Oberkochen, Germany). Samples were dispersed in THF at a concentration of 2 g L^−1^. The unfiltered solution was dropped on a carbon coated copper grid (mesh 200, Science Services, Munich). Electron acceleration voltage was set to 80 kV. Micrographs were taken with a MegaView III/iTEM image acquiring and processing system from Olympus Soft Imaging Systems (OSIS, Münster, Germany) and an Orius 830 SC200W/DigitalMicrograph system from Gatan (Munich, Germany). Particles size measurements were done with “ImageJ” image processing software developed by Wayne Rasband (National Institutes of Health, USA).

Scanning electron microscopy (SEM) micrographs were taken on a Zeiss LEO 1530 GEMINI. The acceleration voltage was set to 3 kV and the sample was sputter-coated with a 1.3 nm platinum layer.

Dynamic light scattering (DLS) measurements were done with an AntonPaar Litesizer 500 in quartz glass cuvettes from Helma at 25 °C in backscattering mode (175°). One measurement consists of six consecutive runs. Samples were dispersed in THF at a concentration of 2 g L^−1^. The unfiltered solution was used. A cumulant fit was used for fitting the experimental data.

Room temperature powder X-ray diffraction (PXRD) data were collected with a STOE StadiP X-ray diffractometer in transmission geometry between 5° and 30° 2*Θ* for all samples, which were placed on flat surfaces. Cu-*K*_α1_ radiation (*λ* = 1.541 Å) was used for the measurements together with a Mythen 1K detector.

For elemental analysis, the carbon, nitrogen and hydrogen contents were determined with a Vario EL III (Elementar Analysensysteme GmbH) with acetanilide as standard or at a Unicube (Elementar Analysensysteme GmbH) with sulfanilamide as standard. The samples were placed in tin boats and measured at least twice. The average of the measurements was used.

Transmission infrared spectra (IR) were collected on a Perkin Elmer Spectrum 100 FT-IR (ATR). The samples were measured directly as solids.

### Computation setting

Theoretical structure calculations on the zinc(ii) precursor complexes and coordination polymer/network models have been performed through density-functional theory (DFT) methods using the ORCA program package.^64^ For all optimizations triple-ξ-valence TZVP^65^ basis sets were used with the generalized gradient approximated functional BP86.^66^ Grimme's third generation D3 correction of dispersion was used.^67,68^ Medium effects were included in a dielectric continuum approach (COSMO), parameterized for acetonitrile;^69^ the inclusion of a stationary dielectric background proved beneficial for the match between experimental and theoretically observed structures. Optimized structures have been identified as stationary points through the absence of imaginary modes in harmonic frequency calculations; spurious low-frequency imaginary modes in some calculations due to –CH_3_ rotations are typical artefacts of DFT-based numerical frequency scans. Coordinates of the computed structures are assembled in the ESI, Tables S1–S4.† Graphical presentation of the vibrational modes are also available (anim_1–6.gif).

#### Phenomenological approach

The input structure of [Zn(TFA)_2_(OH_2_)_4_] was extracted from the available XRD data.^70^ In order to reduce computational cost, we have approximated the coordination polymers and the bulk [Zn(OAc)_2_]·2H_2_O precursor as truncated model complexes. Thereby we have put emphasis on the conservation of the first coordination sphere of the zinc centres. The bidentate bridging ligands were mimicked as monodentate pyridine ligands. In particular we employed the settings:[Zn(TFA)_2_(bppa)_2_]_*n*_ ⇐ [Zn(TFA)_2_(py)_4_][Zn(OAc)_2_(bipy)_2_]_*n*_ ⇐ [Zn_2_(OAc)_4_(py)_4_][Zn(OAc)_2_(OH_2_)_2_] ⇐ [Zn(OAc)_2_(OH_2_)_2_]_5_

The highly H-bonded nature of the molecular modules in [Zn(OAc)_2_]·2H_2_O made it necessary to extract a pentanuclear motif from the crystal structure. Herein the central module possesses a conserved H-bond network (C=O and coordinated water) to serve as the theoretical probe; H-bond donor and acceptor sites of the terminal modules remained unsaturated. As a matter of fact, this model gives very satisfying agreement with the experimental IR spectrum. Graphical representations of the DFT optimized structure models are given in the ESI, Fig. S14 and S15.†

### Synthesis

#### Synthesis of [Zn(TFA)_2_]·H_2_O

[Zn(TFA)_2_]·H_2_O was synthesised by dissolving 1 g (12.3 mmol, 1 eq.) ZnO in 10 mL EtOH and 1.9 mL (2.8 g, 24.6 mmol, 2 eq.) TFA in an ice bath and stirred until complete dissolution. The solution was filtered and the solvent was evaporated on a heating plate at 120 °C for several days. The resulting white powder was transferred into a Schlenk flask, dried *in vacuo* and stored under argon. Yield: 3.42 g (11.7 mmol, 90%). Elemental anal. (%) calc: C 16.49, H 0.65, found: C 16.51, H 0.70.

#### Synthesis of [Zn(OAc)_2_(bipy)]_*n*_ nanocomposites (samples 1–4)

50 mg of the diblock copolymer SV-42 were placed and dissolved in a 50 mL flask in 20 mL THF under reflux until complete dissolution. The polymer solution was cooled down to rt, 2.2 mg (10 μmol, 1 eq.) [Zn(OAc)_2_]·2H_2_O were added and the solution was refluxed for 1 h. Subsequently, the reaction mixture was cooled down to rt and 2.4 mg (15 μmol, 1.5 eq.) 4,4′-bipyridine were added to the solution which was refluxed again for 1 h. At this point, the synthesis can be stopped by removal of the solvent by rotary evaporation to obtain sample 1 (1 cycle). Alternatively, 2.2 mg [Zn(OAc)_2_]·2H_2_O and 2.4 mg 4,4′-bipyridine can be added simultaneously up to 4 more times (samples 2–4; 3–5 cycles). All resulting light yellow solids were dried *in vacuo*.

Elemental anal. (%) found:

Sample 1: C 68.85, H 7.96, N 4.17.

Sample 2: C 65.98, H 7.06, N 4.50.

Sample 3: C 68.61, H 6.92, N 5.38.

Sample 4: C 71.02, H 6.67, N 5.68.

#### Synthesis of [Zn(TFA)_2_(bppa)_2_]_*n*_ nanocomposites (samples 5–8)

50 mg of the diblock copolymer SV-15 were placed in a 50 mL flask fitted with a magnetic stir bar. 20 mL THF were added and the polymer was dissolved under reflux until complete dissolution. The polymer solution was cooled down to rt and 2.0 mg (6.5 μmol, 1 eq.) [Zn(TFA)_2_]·H_2_O were added and the solution was refluxed again for 1 h. Subsequently, the reaction solution was cooled down to rt. 2.8 mg (14 μmol, 2.2 eq.) 1,3-di(4-pyridyl)propane (bppa) were dissolved in 10 mL THF and the solution was added dropwise over 15 min. After the addition of the ligand solution, the reaction mixture was refluxed again for 1 h. The synthesis can be stopped by removal of the solvent by rotary evaporation to obtain sample 5 (1 cycle). Alternatively, the synthesis procedure can be repeated exactly as before to synthesise sample 7 (2 cycles). The resulting light yellow solids were dried *in vacuo*.

Besides the adjustment of the reactants, the synthetic procedure for samples 6 and 8 using the diblock copolymer SV-42 is identical to samples 5 and 7, respectively. 5.8 mg (19 μmol, 1 eq.) [Zn(TFA)_2_]·H_2_O and 8.3 mg (42 μmol, 2.2 eq.) 1,3-di(4-pyridyl)propane were used during the synthesis.

Elemental anal. (%) found:

Sample 5: C 84.08, H 7.66, N 2.42.

Sample 6: C 70.81, H 8.26, N 4.82.

Sample 7: C 67.24, H 7.36, N 2.64.

Sample 8: C 64.40, H 7.02, N 5.05.

## Conflicts of interest

There are no conflicts to declare.

## Supplementary Material

NA-002-D0NA00334D-s001

NA-002-D0NA00334D-s002

NA-002-D0NA00334D-s003

NA-002-D0NA00334D-s004

NA-002-D0NA00334D-s005

NA-002-D0NA00334D-s006

NA-002-D0NA00334D-s007
