# Supplementary figures and images for: Synthesis of Zn-based 1D and 2D coordination polymer nanoparticles in block copolymer micelles
Source: Nanoscale Adv. 2020 Sep 8;2(10):4557–65. doi: 10.1039/d0na00334d (PMC9418959; doi:10.1039/d0na00334d)

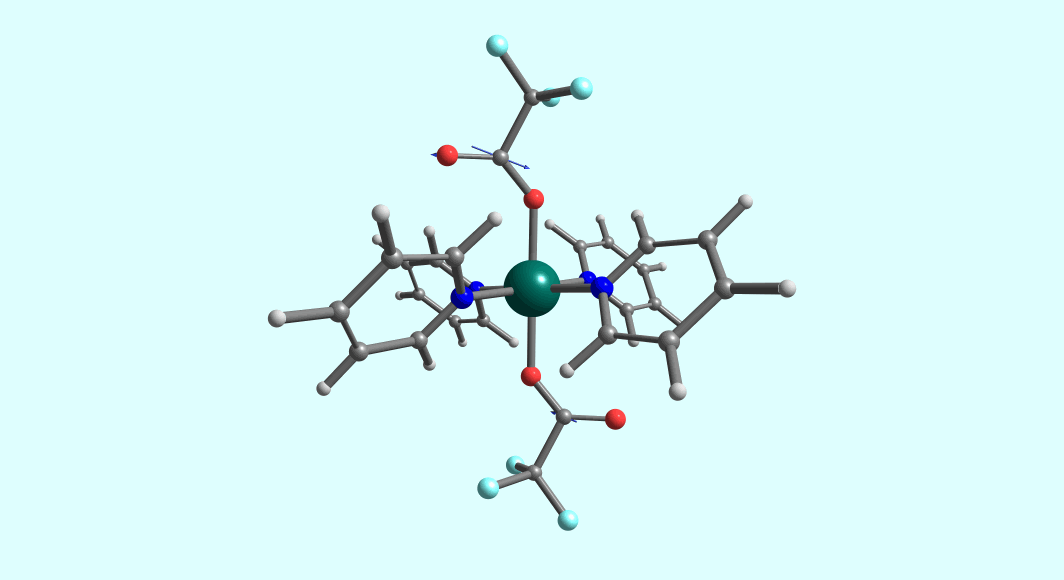

Supplement: NA-002-D0NA00334D-s001 [file NA-002-D0NA00334D-s001.gif]

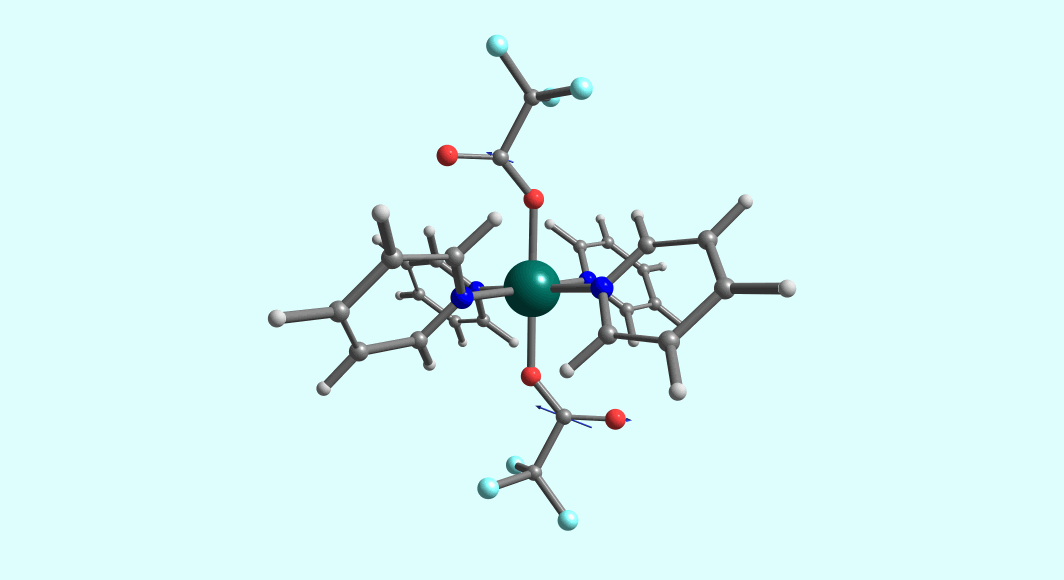

Supplement: NA-002-D0NA00334D-s002 [file NA-002-D0NA00334D-s002.gif]

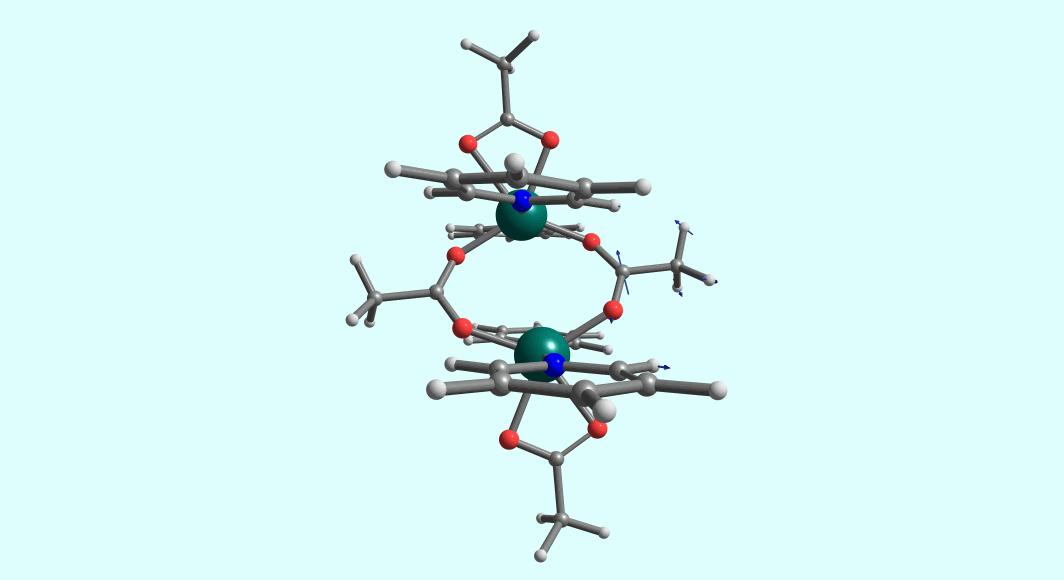

Supplement: NA-002-D0NA00334D-s003 [file NA-002-D0NA00334D-s003.gif]

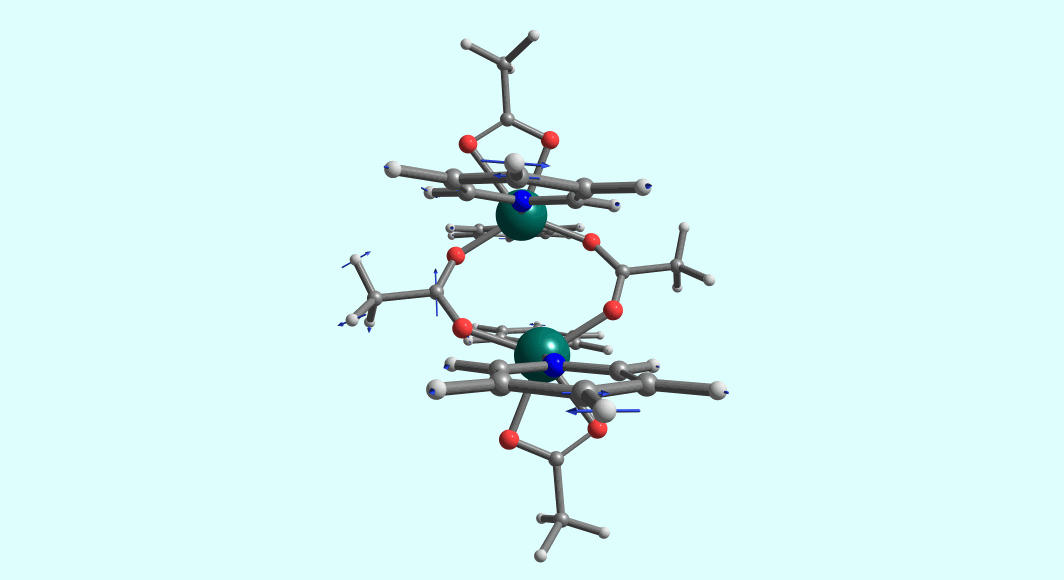

Supplement: NA-002-D0NA00334D-s004 [file NA-002-D0NA00334D-s004.gif]

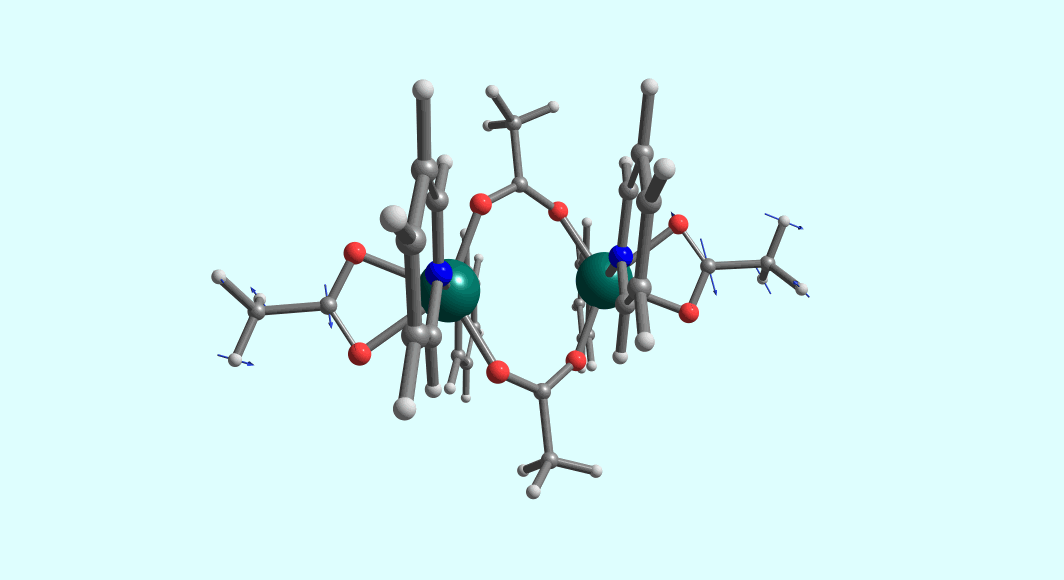

Supplement: NA-002-D0NA00334D-s005 [file NA-002-D0NA00334D-s005.gif]

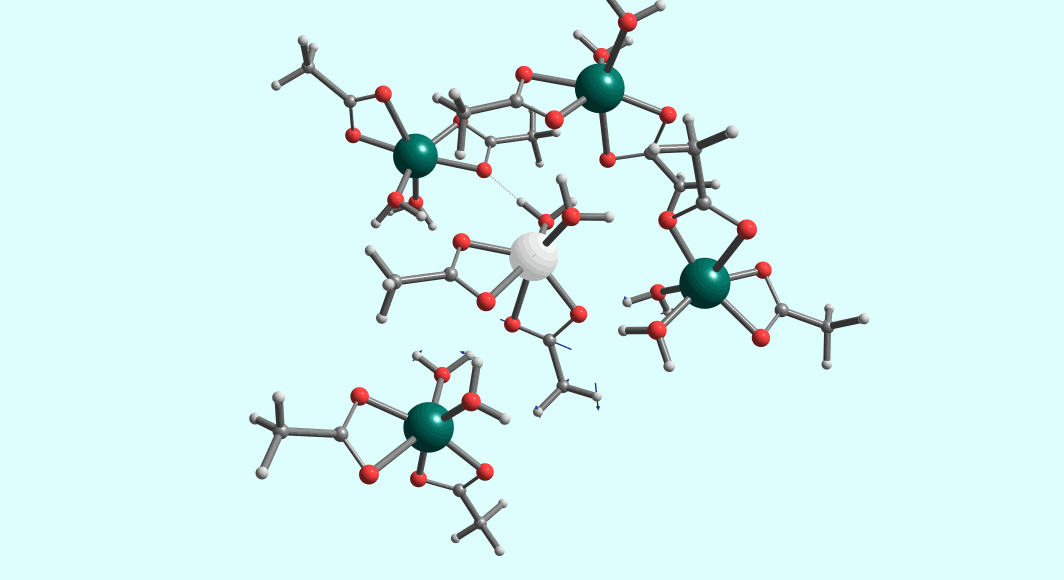

Supplement: NA-002-D0NA00334D-s006 [file NA-002-D0NA00334D-s006.gif]
