# Supplementary material for: Synthesis of Zn-based 1D and 2D coordination polymer nanoparticles in block copolymer micelles
Source: Nanoscale Adv. 2020 Sep 8;2(10):4557–65. doi: 10.1039/d0na00334d (PMC9418959; doi:10.1039/d0na00334d)
Supplement: NA-002-D0NA00334D-s007 [file NA-002-D0NA00334D-s007.pdf]

Electronic Supplementary Material (ESI) for Nanoscale Advances.
This journal is © The Royal Society of Chemistry 2020

Supporting information

Synthesis of Zn-based 1D and 2D coordination polymer nanoparticles in block copolymer micelles

Christoph Göbel,[a] Gerald Hörner, [a]Andreas Greiner,[b] Holger Schmalz,*[b] Birgit Weber*[a]

[a] Department of Chemistry, Inorganic Chemistry IV, Universität Bayreuth, Universitätsstraße 30, 95447 Bayreuth, Germany

[b] Department of Chemistry, Macromolecular Chemistry II and Keylab Synthesis and Molecular Characterization, Bavarian Polymer Institute, Universität Bayreuth, Universitätsstraße 30, 95447 Bayreuth, Germany

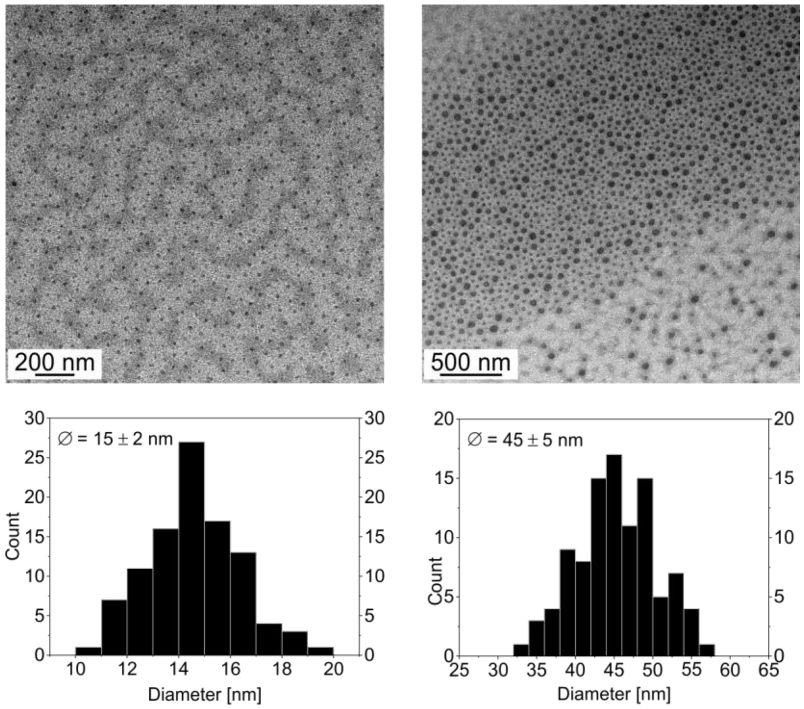

Fig. S1 TEM images of the empty SV-15 (top left) and SV-42 (top right) BCP micelles and the corresponding core size distributions (bottom row).

DLS of empty BCP micelles

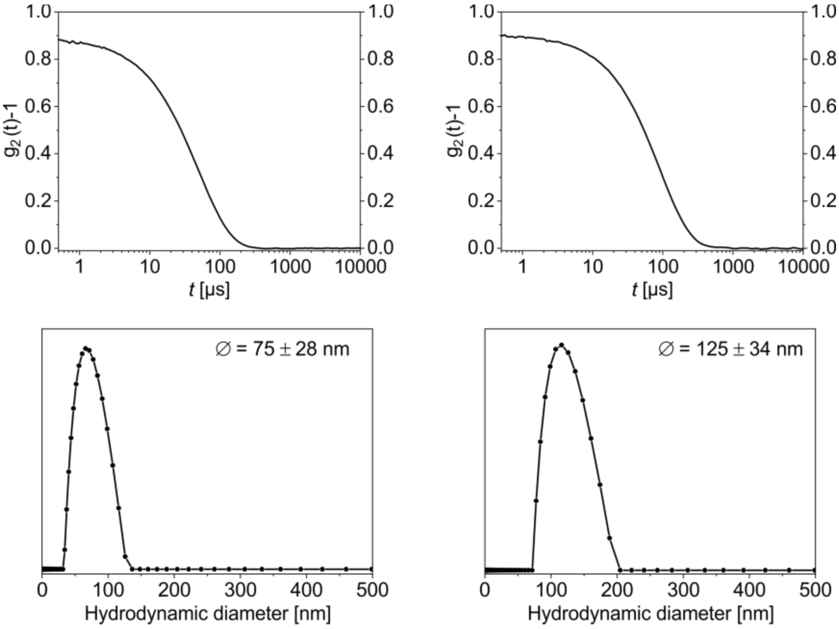

Fig. S2 DLS measurements of the empty SV-15 (left) and SV-42 (right) BCP micelles. The autocorrelation functions g_2(t)-1 *vs*. t are given in the top row and the hydrodynamic diameter distributions are given in the bottom row, respectively.

SEM of a $[Zn(TFA)_2(bppa)_2]_n$ composite with microcrystals

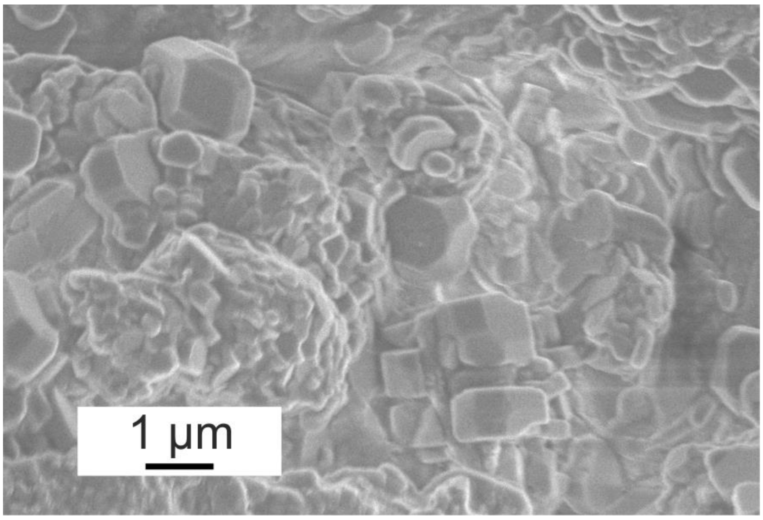

Fig. S31 SEM image of a $[Zn(TFA)_2(bppa)_2]_n$ composite showing truncated cuboctahedron crystals of the CP on the sample surface.

TEM of $[Zn(OAc)_2(bipy)]_n$ nanocomposites (samples 1 – 3)

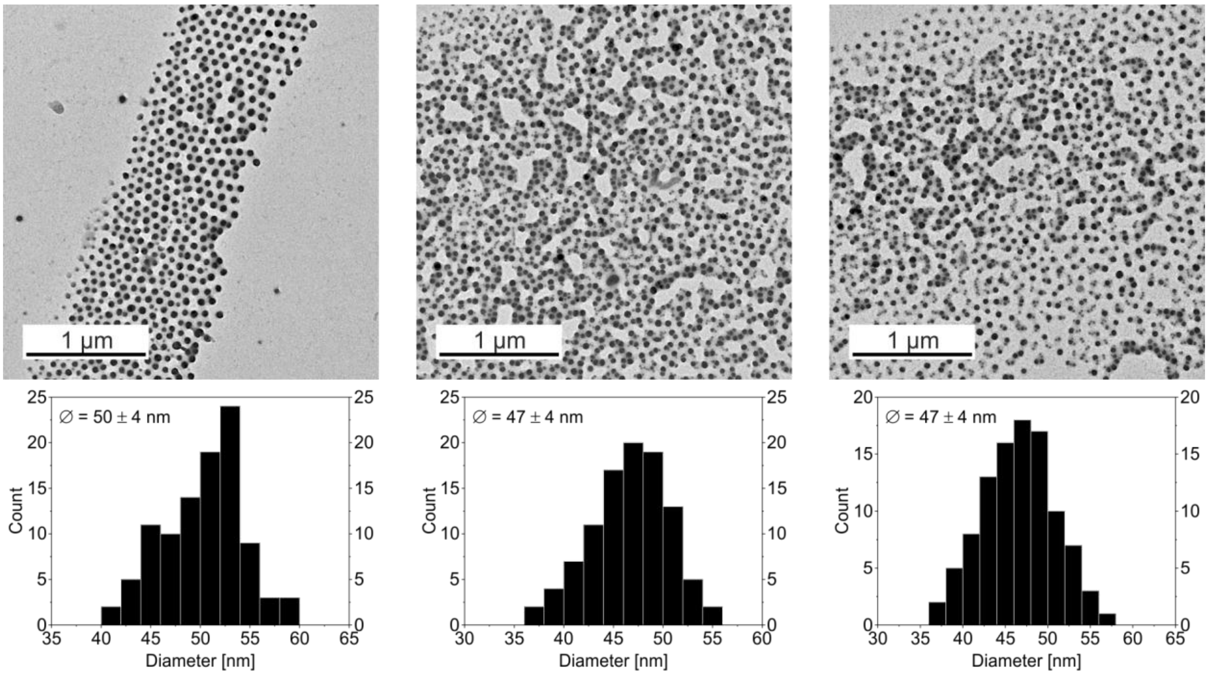

Fig. S4 TEM images (top row) of the $[Zn(OAc)_2(bipy)]_n$ nanocomposite samples **1** (left), **2** (middle) and **3** (right) and the corresponding core size distributions (bottom row).

DLS of $[Zn(OAc)_2(bipy)]_n$ nanocomposites (samples 1 – 3)

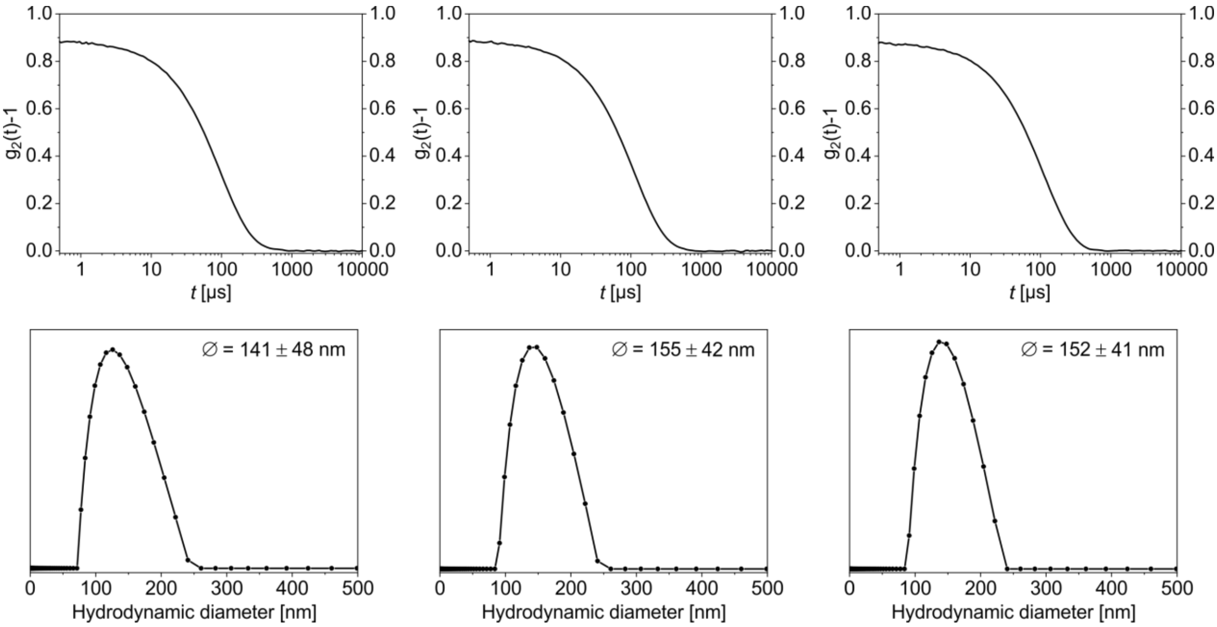

Fig. S5 DLS measurements of the $[Zn(OAc)_2(bipy)]_n$ nanocomposite samples **1** (left), **2** (middle) and **3** (right). The autocorrelation functions $g_2(t)$-1 *vs*. t are given in the top row and the hydrodynamic diameter distributions of the three samples are given in the bottom row, respectively.

DLS of $[Zn(OAc)_2(bipy)]_n$ nanocomposite sample 4

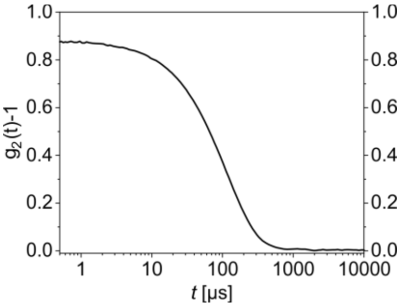

Fig. S6 Autocorrelation function $g_2(t)$-1 *vs*. t of $[Zn(OAc)_2(bipy)]_n$ nanocomposite sample **4**.

SEM of $[Zn(OAc)_2(bipy)]_n$ nanocomposites (samples 1 – 4)

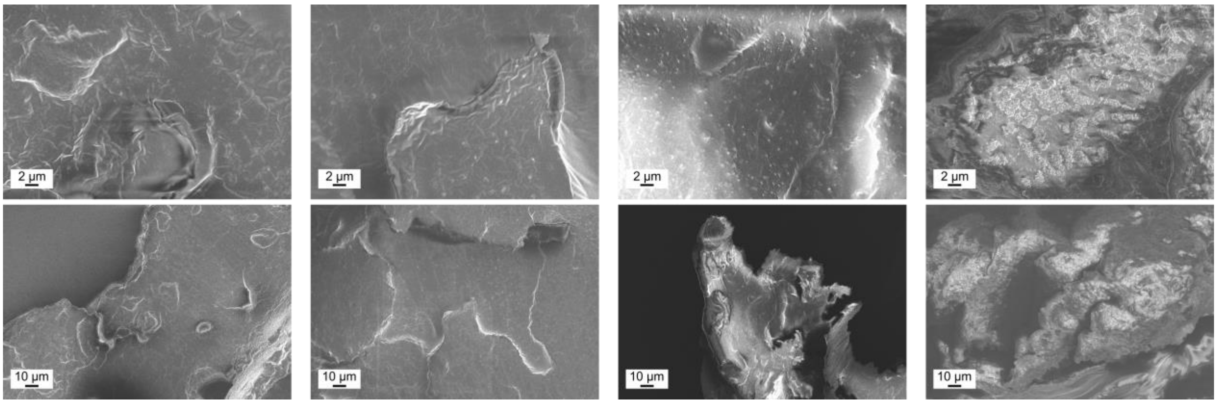

Fig. S7 SEM images of the $[Zn(OAc)_2(bipy)]_n$ nanocomposite samples **1** – **4** (from left to right), showing the absence of microcrystals on the surface of the polymer.

FT-IR of $[Zn(TFA)_2(bppa)_2]_n$ nanocomposites (samples 5 – 8), starting material $[Zn(TFA)_2] \cdot H_2O$ and bulk material $[Zn(TFA)_2(bppa)_2]_n$

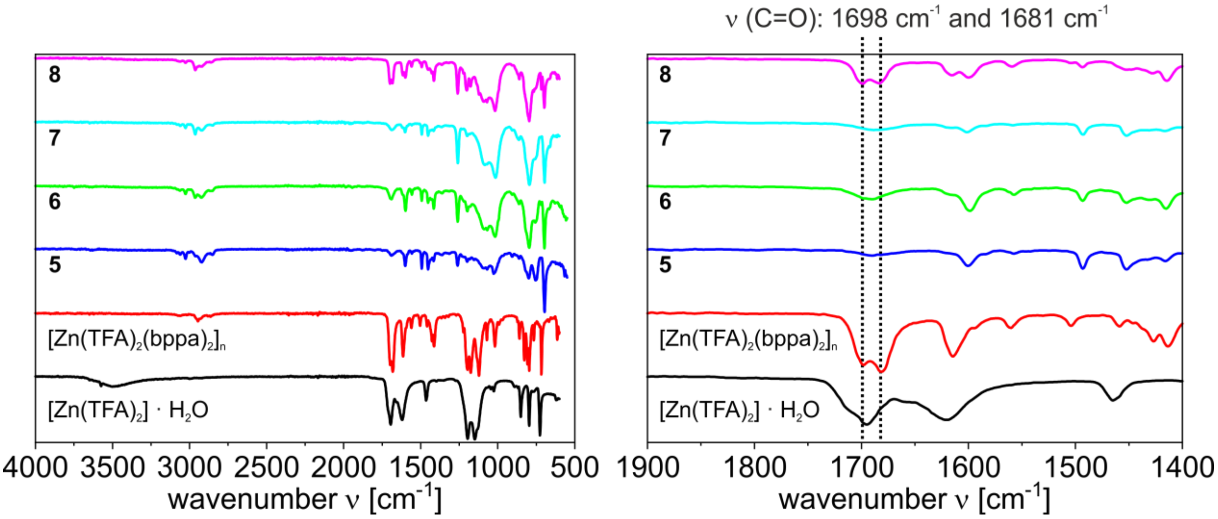

Fig. S8 IR spectra of the starting material $[Zn(TFA)_2] \cdot H_2O$, the bulk material $[Zn(TFA)_2(bppa)_2]_n$ and the $[Zn(TFA)_2(bppa)_2]_n$ nanocomposite samples **5** – **8** in the spectral region of 4000 cm^{-1} to 550 cm^{-1} (left). Each material shows a C=O band at 1698 cm^{-1}. This band is increasing in intensity compared to other bands in the samples **5** – **8** with higher cycle count. Additionally, the bulk material and sample **8** show a second C=O band at 1681 cm^{-1}. A detailed view on the spectral region of 1900 cm^{-1} to 1400 cm^{-1} is also given (right).

TEM of $[Zn(TFA)_2(bppa)_2]_n$ nanocomposites (samples 5 and 6)

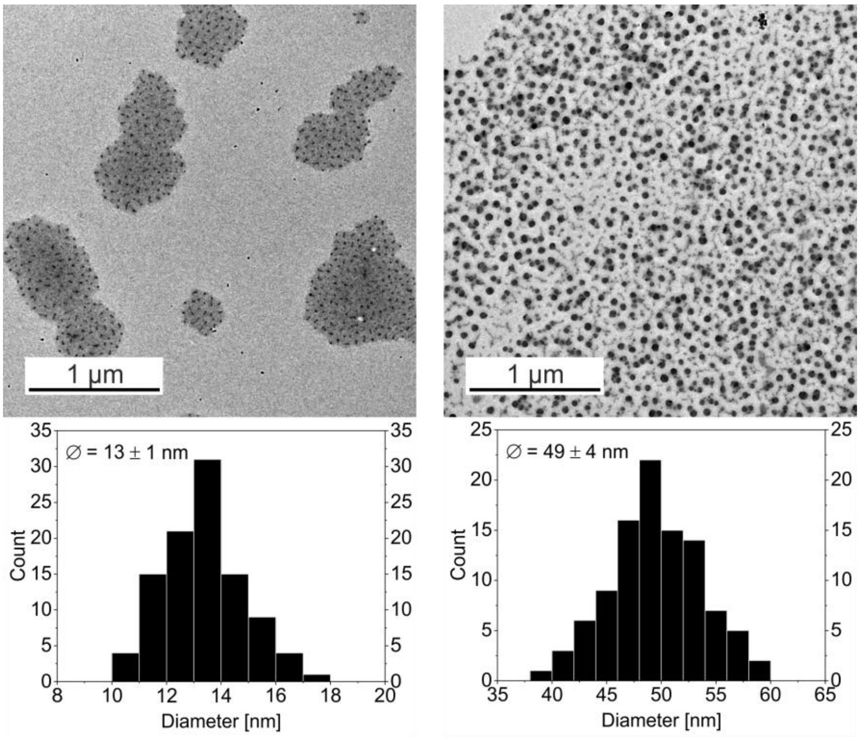

Fig. S9 TEM images (top row) of the $[Zn(TFA)_2(bppa)_2]_n$ nanocomposite sample **5** (left) and sample **6** (right) with the corresponding core size distributions (bottom row).

DLS of $[Zn(TFA)_2(bppa)_2]_n$ nanocomposites (samples 5 and 6)

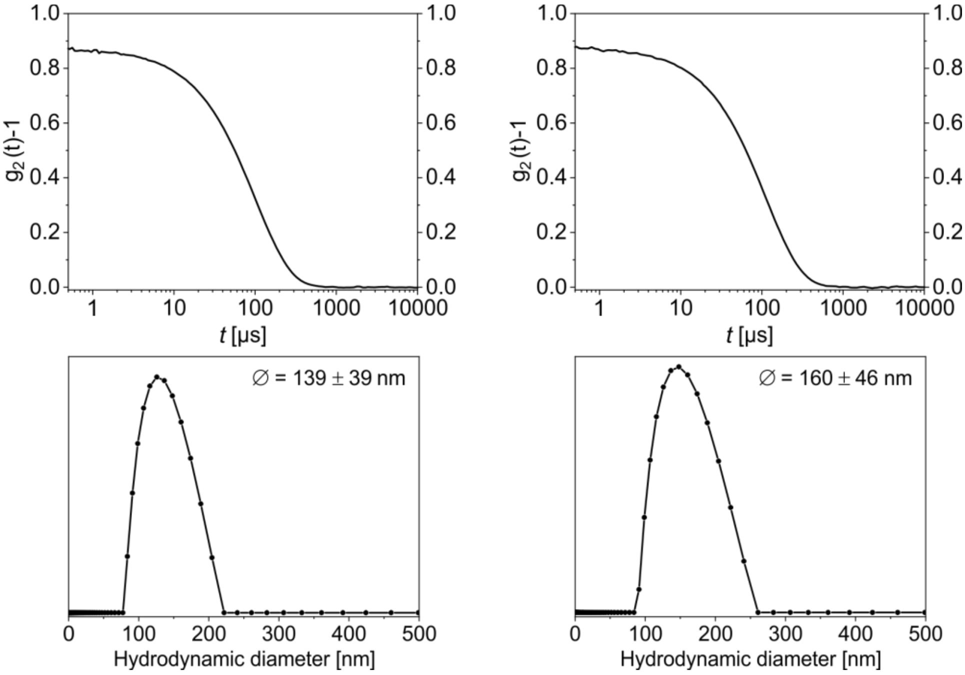

Fig. S10 DLS measurements of the $[Zn(TFA)_2(bppa)_2]_n$ nanocomposite sample **5** (left) and sample **6** (right). The autocorrelation functions $g_2(t)$-1 *vs*. *t* (top) are given together with the hydrodynamic diameter distributions (bottom), respectively.

DLS of $[Zn(TFA)_2(bppa)_2]_n$ nanocomposites (samples 7 and 8)

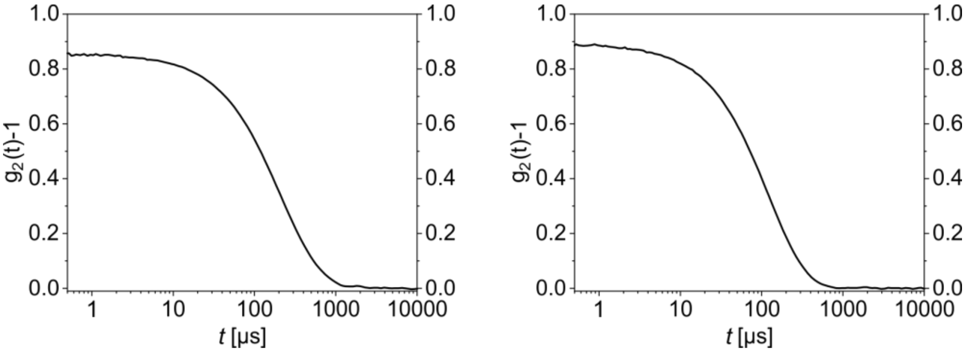

Fig. S11 DLS autocorrelation functions $g_2(t)$-1 *vs*. *t* for the $[Zn(TFA)_2(bppa)_2]_n$ nanocomposite sample **7** (left) and sample **8** (right).

TEM image of $[Zn(TFA)_2(bppa)_2]_n$ nanocomposite particles showing a chain-like structure

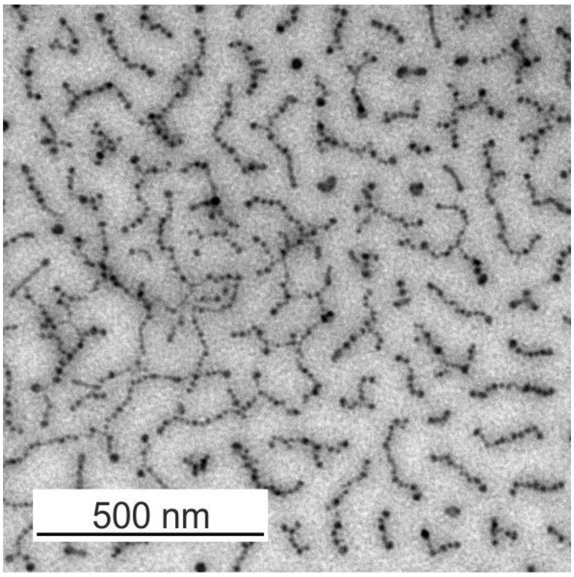

Fig. S12 TEM image of a $[Zn(TFA)_2(bppa)_2]_n$ nanocomposite showing spherical nanoparticles agglomerating into a chain-like structure.

SEM of $[Zn(TFA)_2(bppa)_2]_n$ nanocomposites (samples 5 and 6)

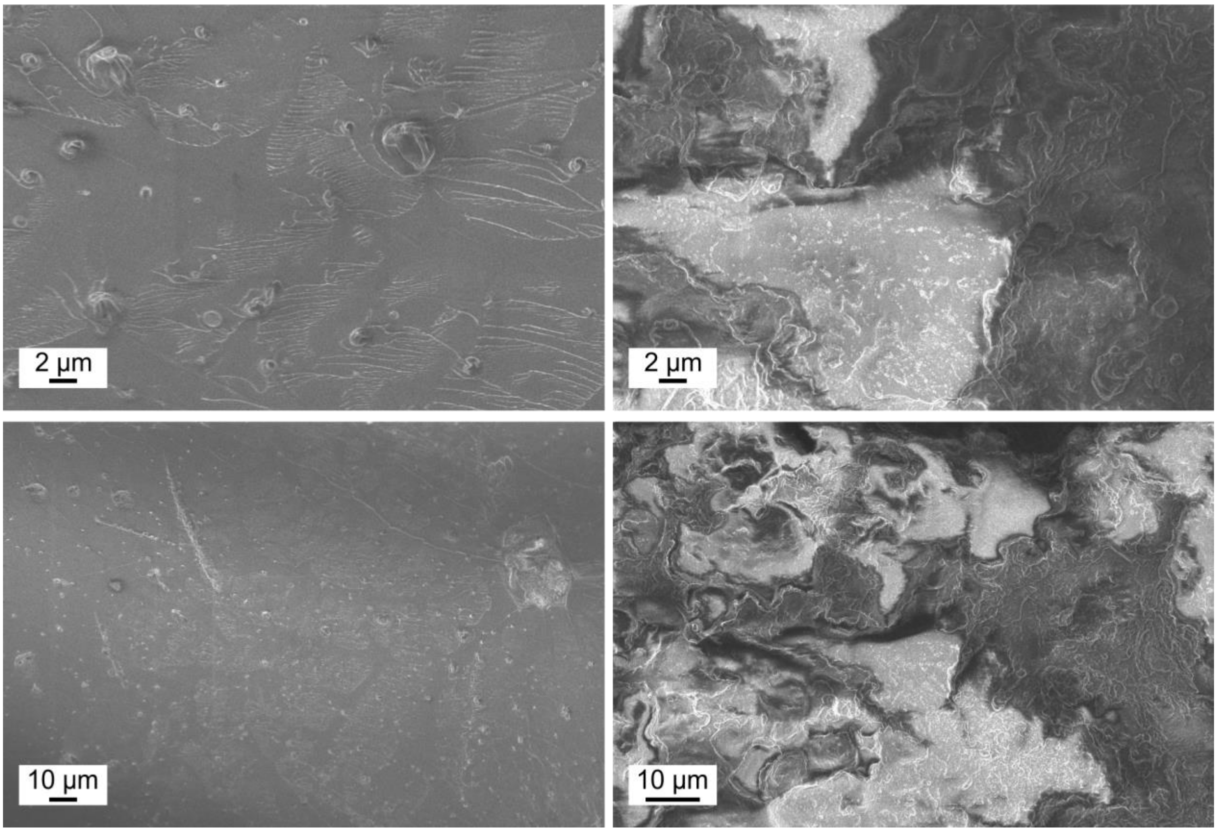

Fig. S13 SEM images of the surfaces of the $[Zn(TFA)_2(bppa)_2]_n$ nanocomposite sample **5** (left) and **6** (right), showing the absence of microcrystals.

Table S1 Cartesian coordinates of optimized mononuclear $[Zn(TFA)_2(py)_4]$.

**Zn**	-0.276558	2.13155	-0.688121
**C**	-2.558684	2.321083	1.34992
**C**	-3.634943	1.947722	2.152391
**H**	-4.126515	2.689182	2.782108
**C**	-4.06365	0.617259	2.126218
**C**	-3.396773	-0.289531	1.298695
**C**	-2.328578	0.162712	0.524653
**N**	-1.916161	1.446384	0.551077
**H**	-4.904818	0.293244	2.740859
**H**	-3.697914	-1.335982	1.247617
**C**	-1.325065	5.107707	-1.324931
**C**	-2.680554	5.826038	-1.669412
**O**	-1.504187	3.886117	-1.042195
**O**	-0.290773	5.795446	-1.356083
**F**	-3.514455	5.83803	-0.58362
**F**	-3.34782	5.189126	-2.677001
**F**	-2.520068	7.119454	-2.05771
**O**	0.948072	0.38585	-0.282998
**C**	0.899188	-0.86024	-0.504605
**F**	2.575286	-1.504364	1.128004
**O**	-0.033477	-1.584279	-0.890865
**C**	2.278471	-1.556079	-0.208062
**F**	2.304245	-2.867454	-0.566749
**F**	3.306825	-0.943911	-0.866857
**H**	-2.193003	3.347177	1.321902
**H**	-1.765515	-0.505185	-0.133403
**N**	1.336027	2.837767	-1.937289
**H**	0.02806	3.615491	-3.342848
**C**	1.080161	3.491539	-3.086259
**H**	2.767066	2.098145	-0.640641
**C**	2.618426	2.648793	-1.569005
**C**	2.091016	3.981129	-3.909787
**C**	3.688449	3.11057	-2.3337
**C**	3.421095	3.78947	-3.525942
**H**	1.835119	4.502036	-4.832005
**H**	4.710714	2.935822	-1.998467
**H**	4.236617	4.16198	-4.147007
**H**	-2.933268	-0.358319	-5.511315
**H**	-0.487621	-0.74814	-5.048261
**C**	-2.4503	0.096526	-4.645458
**C**	-1.093617	-0.122938	-4.392552
**C**	-3.177244	0.911292	-3.772627
**H**	-4.23595	1.112887	-3.935328
**C**	-0.516221	0.475802	-3.27481
**H**	0.538265	0.336595	-3.036538
**C**	-2.522687	1.475067	-2.678918
**N**	-1.21524	1.259285	-2.431962
**H**	-3.033678	2.132821	-1.975917
**H**	0.889926	4.843032	0.20706
**N**	0.669849	2.971744	1.072421
**C**	1.080579	4.256022	1.109497
**C**	0.865196	2.193684	2.155951
**H**	0.52628	1.162019	2.06669
**C**	1.69735	4.805904	2.233376
**H**	2.013104	5.848952	2.222128
**C**	1.474964	2.66792	3.315821
**C**	1.899086	3.999282	3.356466
**H**	1.614264	2.002657	4.16792
**H**	2.379917	4.400631	4.249703

Table S2 Cartesian coordinates of optimized mononuclear $[Zn(TFA)_2(OH_2)_4]$.

**Zn**	-0.199982	2.219365	-0.819565
**C**	-2.437158	3.974124	-1.711991
**C**	-3.118371	5.383708	-1.658205
**O**	-1.363785	3.928002	-1.031129
**O**	-2.9979	3.091999	-2.390561
**F**	-2.279444	6.345047	-2.139725
**F**	-3.433185	5.72459	-0.375359
**F**	-4.259515	5.441048	-2.384408
**O**	0.880295	0.501668	-0.317929
**C**	0.610443	-0.659197	-0.763656
**F**	1.448851	-1.848981	1.163788
**O**	-0.277888	-1.026652	-1.556466
**C**	1.569144	-1.758962	-0.192398
**F**	1.32034	-2.988172	-0.703597

**F**	2.870924	-1.46113	-0.465698
**O**	1.378556	2.990389	-2.088653
**O**	-0.97931	1.354651	-2.574225
**O**	0.76385	3.132065	0.856188
**H**	1.148309	2.724078	-2.99955
**H**	2.254866	2.593604	-1.927477
**H**	-1.900113	1.738023	-2.607127
**H**	-1.005062	0.386665	-2.336373
**H**	1.097118	4.03721	0.714768
**H**	1.525826	2.619938	1.185344
**H**	-1.455476	0.838923	1.167272
**H**	-2.195995	2.184962	1.006609
**O**	-1.773578	1.469986	0.495266

Table S3 Cartesian coordinates of optimized binuclear [$Zn_2(OAc)_4(py)_4$].

**Zn**	-1.040115	2.513125	-0.382913
**C**	-2.96243	1.875928	1.916033
**C**	-4.134951	1.494851	2.567773
**H**	-4.152581	1.425702	3.655584
**C**	-5.269548	1.210607	1.804093
**C**	-5.191061	1.325613	0.413793
**C**	-3.983273	1.71735	-0.161824
**N**	-2.88689	1.986713	0.575294
**H**	-6.201922	0.910837	2.284923
**H**	-6.053171	1.119917	-0.220738
**C**	-2.116779	4.472185	-2.532806
**C**	-2.773595	4.505906	-3.902184
**O**	-2.219648	3.398193	-1.864961
**O**	-1.539702	5.537509	-2.147051
**H**	-3.804072	4.877354	-3.787503
**H**	-2.821974	3.500561	-4.336463
**H**	-2.241607	5.19167	-4.572485
**O**	-0.118666	0.618096	0.222624
**C**	-0.49666	-0.005734	-0.830268
**H**	-0.844955	-2.058756	-0.31609
**O**	-1.160765	0.576392	-1.74323
**C**	-0.160394	-1.481965	-0.956534
**H**	-0.274406	-1.829548	-1.990281
**H**	0.861934	-1.67791	-0.606167
**H**	-2.047497	2.121448	2.455141
**H**	-3.864027	1.843107	-1.237368
**N**	0.727106	3.092937	-1.480848
**H**	-0.233699	2.708939	-3.27163
**C**	0.694219	3.069126	-2.829138
**H**	1.85829	3.468648	0.212487
**C**	1.861236	3.490497	-0.875332
**C**	1.773496	3.468255	-3.612598
**C**	2.986207	3.907784	-1.58462
**C**	2.93991	3.90701	-2.979376
**H**	1.695647	3.437278	-4.699437
**H**	3.876862	4.230122	-1.046522
**H**	3.798987	4.237735	-3.564046
**H**	-5.696923	4.506213	2.811207
**H**	-5.792694	4.57468	0.295651
**C**	-4.851112	4.874377	2.229801
**C**	-4.906976	4.906796	0.835845
**C**	-3.693585	5.331081	2.866621
**H**	-3.610323	5.33908	3.953461
**C**	-3.797708	5.369292	0.130142
**H**	-3.800846	5.417363	-0.956798
**C**	-2.630992	5.778233	2.086707
**N**	-2.671043	5.78305	0.738308
**H**	-1.710544	6.154208	2.531562
**Zn**	-0.899269	6.353721	-0.347928
**H**	0.104237	4.690391	3.906177
**H**	-2.88528	10.440265	0.342268
**O**	-1.827178	8.279209	-0.999132
**O**	-0.433894	3.348217	1.407383
**O**	0.28441	5.475333	1.137303
**C**	0.183202	4.390372	1.789039
**C**	-1.433592	8.879969	0.058689
**C**	0.835138	4.340433	3.160139
**C**	-1.793617	10.344863	0.23991
**O**	-0.763442	8.27368	0.954519
**H**	1.119525	3.314394	3.421208
**H**	-1.498757	10.917626	-0.651125
**H**	1.705015	5.006294	3.202147
**H**	-1.311181	10.770675	1.127254
**H**	0.160066	6.631741	-3.209967
**N**	0.952176	6.847868	-1.317088
**C**	1.060466	6.901399	-2.658548
**C**	2.030619	7.151074	-0.565864
**H**	1.884325	7.073591	0.510861
**C**	2.247496	7.257683	-3.298217
**H**	2.290634	7.280232	-4.387279
**C**	3.251508	7.51875	-1.128899
**C**	3.36328	7.575124	-2.520706
**H**	4.098034	7.752546	-0.483299
**H**	4.306923	7.855114	-2.991314

Table S4 Cartesian coordinates of optimized pentanuclear $[Zn(OAc)_2(OH_2)_2]_5$.

**Zn**	5.726065	0.494003	8.327941
**C**	7.576767	2.061351	7.338346
**O**	7.329943	2.275238	8.561747
**O**	6.942583	1.102215	6.738581
**C**	8.562674	2.904475	6.58257
**O**	4.438492	-0.788251	7.453453
**H**	8.804238	2.477104	5.603851
**H**	9.479234	3.012023	7.176843
**H**	8.145813	3.917608	6.447091
**H**	4.72249	-1.014434	6.500523
**H**	4.406257	-1.678066	7.923202
**C**	4.141839	2.400161	9.244153
**O**	4.378142	2.48832	8.009765
**O**	4.590109	1.356521	9.887231
**C**	3.363211	3.446047	9.984888
**O**	6.893206	-0.741045	9.412241
**H**	3.314978	3.234069	11.058328
**H**	2.343323	3.483797	9.575683
**H**	3.812754	4.43848	9.821852
**H**	6.386333	-1.210457	10.153373
**H**	7.270564	-1.489555	8.848251
**Zn**	6.247793	-0.797615	3.210782
**C**	4.769857	-2.457133	4.43252
**O**	5.120717	-2.658191	3.231423
**O**	5.217017	-1.403892	5.041139
**C**	3.823919	-3.382797	5.142773
**O**	7.173139	0.819177	4.143284
**H**	2.864806	-2.864106	5.289876
**H**	3.653864	-4.291828	4.557834
**H**	4.211275	-3.641221	6.135749
**H**	7.040393	0.926808	5.142661
**H**	6.989443	1.682529	3.733616
**C**	8.034098	-1.242865	1.52291
**O**	8.140504	-1.7695	2.680803
**O**	7.019975	-0.505152	1.245944
**C**	9.077077	-1.500526	0.463673
**O**	4.495698	0.328396	2.655941
**H**	9.290131	-0.579866	-0.094676
**H**	9.997967	-1.898719	0.903236
**H**	8.679713	-2.237793	-0.251084
**H**	4.605858	0.988915	1.947703
**H**	4.003741	0.779634	3.365792
**Zn**	4.350915	-0.93309	12.711542
**C**	2.777247	-1.352889	14.591116
**O**	2.844574	-2.211846	13.649791
**O**	3.501032	-0.29099	14.537697
**C**	1.882804	-1.58569	15.782008
**O**	5.438882	0.895884	12.434796
**H**	1.491772	-0.636173	16.166626
**H**	1.059888	-2.263437	15.527825
**H**	2.480734	-2.051965	16.580676
**H**	5.233813	1.588927	13.087954
**H**	5.208776	1.263051	11.539718
**C**	6.3786	-2.445955	12.309049
**O**	6.104527	-2.257003	13.521653
**O**	5.635395	-1.887297	11.39184
**C**	7.523575	-3.308126	11.853612
**O**	2.986926	-0.379035	11.191187
**H**	8.211867	-2.714045	11.234647
**H**	8.065827	-3.730242	12.70531
**H**	7.127656	-4.113954	11.218365
**H**	3.3805	0.319323	10.604158
**H**	2.098017	-0.078456	11.450437
**Zn**	6.386117	-4.548498	8.085357
**C**	7.804019	-2.936456	6.766183
**O**	7.67498	-2.818637	8.049295
**O**	7.260591	-3.931461	6.193439
**C**	8.594842	-1.911402	6.01111
**O**	5.302876	-5.999501	6.982302
**H**	8.533403	-2.069319	4.930095
**H**	9.644913	-1.95798	6.336053
**H**	8.221455	-0.910359	6.264804
**H**	5.712069	-6.269124	6.140037
**H**	5.035959	-6.824538	7.427039
**C**	4.399596	-3.909588	9.511644
**O**	4.616195	-3.231135	8.443049
**O**	5.206657	-4.859403	9.805394
**C**	3.190224	-3.642464	10.356453
**O**	7.916924	-5.812738	8.801265
**H**	3.340347	-3.995439	11.381807
**H**	2.339579	-4.187481	9.918048
**H**	2.951738	-2.573056	10.362591
**H**	7.632825	-6.616835	9.273145
**H**	8.594608	-6.108401	8.165734
**Zn**	6.084227	6.088493	8.057789
**C**	8.248823	6.985807	7.092639
**O**	7.906957	7.50041	8.198146
**O**	7.499802	6.097775	6.524928

**C**	9.542791	7.3679	6.414416
**O**	4.839964	4.82196	6.923366
**H**	10.24172	6.519467	6.477942
**H**	9.997542	8.241923	6.892488
**H**	9.367612	7.568917	5.349024
**H**	5.188761	4.666444	6.028251
**H**	4.60035	3.92058	7.30779
**C**	4.202729	7.54232	8.895012
**O**	4.671409	7.822676	7.748668
**O**	4.705653	6.571988	9.58065

**C**	3.042024	8.31419	9.47475
**O**	7.022274	4.741686	9.365302
**H**	2.154206	7.663994	9.489471
**H**	2.821819	9.207264	8.880314
**H**	3.257687	8.597344	10.513423
**H**	6.644483	4.730826	10.261422
**H**	7.205003	3.78504	9.098501

Graphical representation of DFT optimized structures.

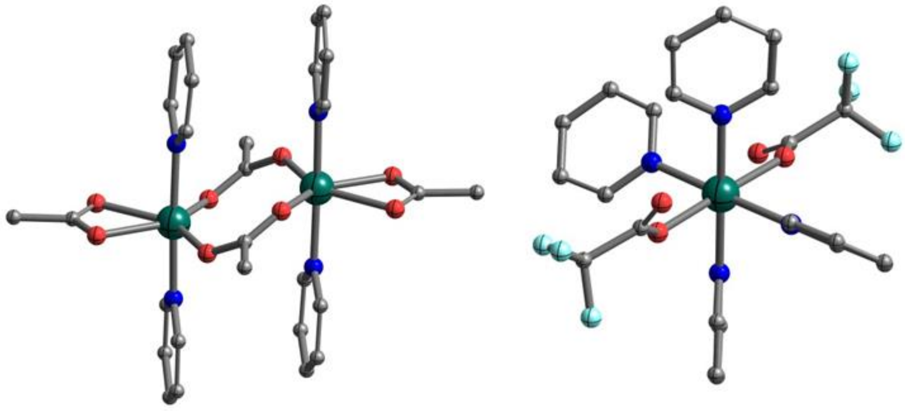

Fig. S14. DFT-optimised structure of CP models; left: $[Zn_2(OAc)_4(py)_4]$ as a model of 1D CP $[Zn(OAc)_2(bipy)_2]_n$; right: $[Zn(TFA)_2(py)_4]$ as a model of 2D CN $[Zn(TFA)_2(bppa)_2]_n$.

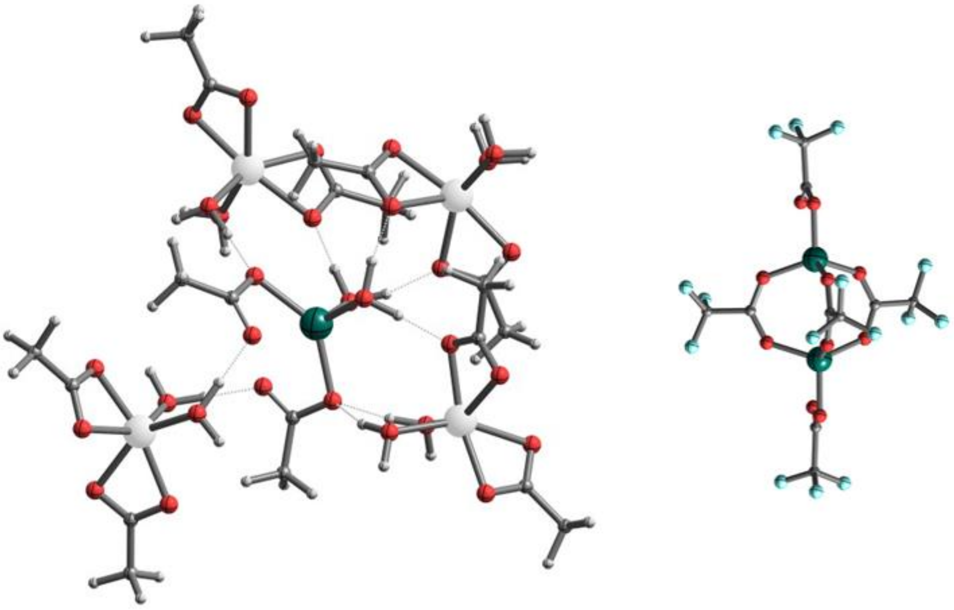

Fig. S15. DFT-optimised structure of precursor models; left: $[Zn(OAc)_2(OH_2)_2]_5$ as a model of bulk $Zn(OAc)_2 \times 2H_2O$ (terminal Zn centres given in white); right: $[Zn_2(TFA)_5]^-$ as a model of $Zn(TFA)_2$.
